# Vitamin D Status Is Not Associated with Outcomes of Experimentally-Induced Muscle Weakness and Pain in Young, Healthy Volunteers

**DOI:** 10.1155/2010/674240

**Published:** 2010-12-19

**Authors:** Susan M. Ring, Erin A. Dannecker, Catherine A. Peterson

**Affiliations:** ^1^Department of Nutrition and Exercise Physiology, 217 Gwynn Hall, University of Missouri-Columbia, Columbia, MO 65211, USA; ^2^Physical Therapy Department, 106 Lewis Hall, University of Missouri-Columbia, Columbia, MO 65211, USA

## Abstract

Vitamin D receptors have been identified in skeletal muscle; and symptoms of vitamin D deficiency include muscle weakness and pain. Moreover, increased serum 25-hydroxyvitamin D (25(OH)D) concentrations have been associated with improved muscle function. To further clarify the importance of vitamin D to muscle, we examined the association between vitamin D status and exercise-induced muscle pain and weakness in healthy people. Muscle damage to the elbow flexors was induced with eccentric exercise (EE) in 48 individuals (22.5 ± 3.2 yrs). Muscle pain ratings following unloaded movement and peak isometric force (IF) were collected before EE and for 4 days post-EE. Linear regression was used to determine if serum 25(OH)D was a predictor of any outcome. In males, *R* ^2^-values from 0.48 to 1.00. *R* ^2^ for IF ranged from 0 to 0.02 and *P*-values from 0.48 to 1.00. In females, *R* ^2^ for pain ratings ranged from 0.01 to 0.11 and *P*-values from 0.14 to 0.59. *R* ^2^ for IF ranged from 0 to 0.04 and *P*-values from 0.41 to 0.90. In conclusion, vitamin D status did not predict muscle pain or strength after EE-induced muscle damage in young healthy men and women.

## 1. Introduction

The prevalence of vitamin D insufficiency in the US, as measured by serum concentrations of 25-hydroxyvitamin D (25(OH)D), has been classified as an “epidemic”—affecting approximately 25%–35% of the adult population [1]. Globally, the prevalence is reported to be even higher, affecting up to 80% of high-risk populations such as dark-skinned individuals or those with limited/no sun exposure [2].

The importance of vitamin D in skeletal muscle (SkM) function has been demonstrated by the presence of the vitamin D receptor (VDR) in both the nucleus and plasma membrane of human SkM fibers [3] and by defective muscle development and function in VDR knockout mice [4]. The biologically active form of vitamin D, 1,25-dihydroxyvitamin D (1,25(OH)_2_D), acts through genomic control to regulate protein synthesis in muscle cells. Rapid responses to 1,25(OH)_2_D in SkM are mediated by the membrane-bound VDR, which triggers several key cell signal cascades, especially those involved in calcium flux and mitogenic processes [5]. 

Reports of severe adult-onset vitamin D deficiency, known as osteomalacic myopathy, provided the first evidence of a link between vitamin D and SkM in humans. In this condition of soft, undermineralized bone, often the first observed symptoms are muscle weakness and pain [6]. Remarkably, muscle weakness can even exist during deficiency without biochemical signs of bone involvement [7]. Several large cross-sectional studies have described diminished muscle strength and physical performance in individuals with lower serum 25(OH)D [8–10]. Moreover, studies have shown that when vitamin D supplementation is sufficient to raise serum 25(OH)D concentrations, outcomes related to muscle function improve risk of falls, relative size and number of type II muscle fibers, and lower limb and grip strength [11, 12].

Vitamin D deficiency is also associated with persistent musculoskeletal pain. Ninety-three percent of ambulatory outpatients with chronic, nonspecific musculoskeletal pain were found to be deficient in vitamin D [13]. In a separate report involving chronic low back pain patients, 83% had low vitamin D status; following vitamin D therapy, 95% of these patients showed improvement in their symptoms [14].

 To further clarify the importance of vitamin D to muscle, the present study examined the association of vitamin D status and exercise-induced muscle pain and weakness in healthy people. The muscle pain and weakness were induced in the elbow flexors by vigorous eccentric exercise. We tested the hypothesis that lower serum concentrations of 25(OH)D are associated with greater muscle weakness and pain after eccentric exercise in young, healthy volunteers.

## 2. Materials and Methods

### 2.1. Participant Recruitment

The relationship between vitamin D status and muscle pain/strength after eccentric exercise (EE) was explored using a secondary analysis of data collected from an investigation of exercise-induced muscle damage that was designed to examine sensitization to pressure and activity after lengthening contractions [15]. The study was approved by the University of Missouri Health Sciences Institutional Review Board; all participants gave informed written consent prior to data collection.

Twenty-seven males and 21 females, aged 18 to 45 years, were recruited by public advertisement from the University of Missouri Columbia campus and community. Exclusion criteria were arm strength training two to three times per week within the previous six months; injury to the shoulder, arm, wrist, or hand in the previous six months; consumption of any performance enhancing drugs or medications that could affect pain perception or response to eccentric exercise; illness within one week of participation; chronic medical conditions affecting pain perception; hypercholesterolemia or thyroid dysfunction; history of heat illness, kidney or liver dysfunction, cardiac condition, or muscle disease; family history of muscle disease or sickle cell disease; allergy to aspirin or nonsteroidal anti-inflammatory medications; lack of regular menstruation for females.

Prior to the initial two sessions, participants were instructed to refrain from smoking for three hours, alcohol for 12 hours, and taking pain medication for 24 hours. After the initial session, five more sessions were conducted, all of which were held during the follicular phase of the menstrual cycle for females. Throughout the five subsequent sessions, self-care behaviors such as massage, consumption of any pain relieving medications, and the application of heat, cold or pain relieving cream, were prohibited.

### 2.2. Study Protocol and Assessments

During the initial session, descriptive data were collected by questionnaire, including sex, race, ethnicity, date of last menstrual period, and medications, including birth control, and dietary supplements consumed. Height and weight were measured. Baseline isometric strength of the nondominant elbow flexors was measured as peak force, using the Biodex System 3 (Biodex Medical Systems, Shirley, NY). Five maximal voluntary isometric contractions at elbow flexion of 90° were performed, each lasting 5 seconds and separated by a 2-minute rest. 

Baseline pain ratings for the nondominant arm were obtained during complete flexion and extension of the elbow in supination. Numerical ratings for both pain intensity and pain unpleasantness were recorded, using a validated scale of 0–100 [16], in which 0 indicated “no pain” or “not at all unpleasant” and 100 meant the “most intense pain sensation imaginable” or “most unpleasant pain imaginable,” respectively. 

At least 24 hours after the first session, the volunteers returned for a second session during which muscle strength and pain measures were repeated, followed by eccentric strength testing of the nondominant arm [17]. Then an EE protocol was completed that consisted of 3 sets of 12 repetitions with strong verbal encouragement to produce 75% of maximal eccentric peak torque on each repetition. A two-minute rest period separated the sets. One hour later, and then daily for 4 days, participants returned for muscle pain and isometric strength measurements. This induced muscle pain has been documented to last up to 10 days [18], while the muscle weakness has been reported to last over 30 days in some participants [19].

### 2.3. Vitamin D Status

Serum 25(OH)D was assayed in serum two days after EE using a commercially available radioimmunoassay (DiaSorin, Stillwater, MN; intra-assay CV = 10.8%).

### 2.4. Data Analysis

Linear regression of all outcome variables against serum 25(OH)D was carried out using the statistical package “*R*” (*The R Foundation for Statistical Computing*, 2.2.1, 2005). There is evidence of sex-specific variations in muscle strength, (as well as other phenotypic traits), according to specific VDR polymorphisms [20, 21]. Thus, data for men and women were analyzed separately. A *P* value  .05 was considered to be statistically significant for all analyses.

## 3. Results

Participant characteristics and serum 25(OH)D are listed in Table 1. According to the recommended vitamin D status classifications of Grant and Holick [22]. 10.4% of all subjects were deficient (<50 nmol/L), 54.2% were insufficient (50–80 nmol/L), and 33.3% were sufficient (80–250 nmol/L); one male participant had a very high 25(OH)D serum concentration (269.5 nmol/L). 

**Figure 1:**
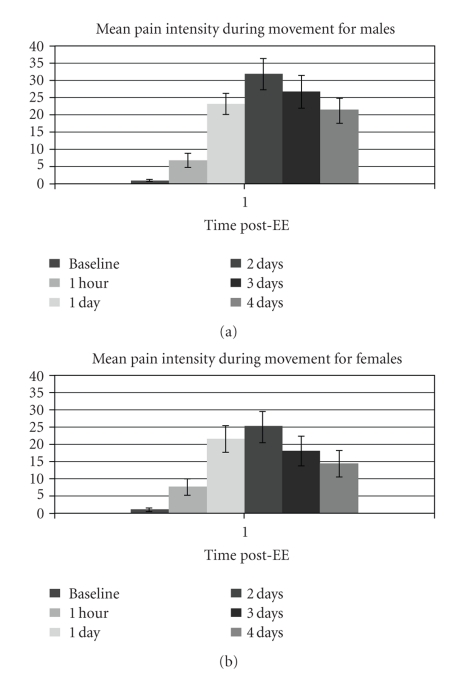
Progress of pain intensity throughout the study period. Mean (±SE) of pain intensity ratings (scale 0–100) during unloaded movement through full range of motion of the elbow. Time after EE refers to time elapsed after the eccentric exercise protocol, during which muscle damage was induced. Pain intensity rating was one of two distinct pain outcomes. The other, pain unpleasantness rating, followed a similar pattern following the eccentric exercise protocol. Males: *N* = 26 (baseline), *N* = 27 (all other times); females: *N* = 20 (4 days), *N* = 21 (all other times).

**Figure 2:**
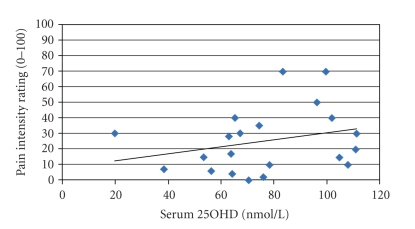
Progress of muscle strength throughout the study period. Mean (±SE) peak torque for males and females during isometric strength testing of the elbow flexors. Time after EE refers to time elapsed after the eccentric exercise protocol, during which muscle damage was induced. *N* m = Newton-meters. Males: *N* = 25 (4 days), *N* = 26 (baseline, 1 hour, 2 and 3 days), *N* = 27 (1 day); females: *N* = 20 (4 days), *N* = 21 (all other times).

As expected, pain measures for both sexes increased after EE, peaking 2 days after EE. Figure 1 depicts the time course for pain intensity during unloaded flexion and extension. This plot is representative of the progression of pain unpleasantness as well. Also as expected, peak torque for both sexes decreased after EE, reaching a nadir at 1 hour for females and 2 days for males, and then gradually increasing, as shown in Figure 2.

**Figure 3:**
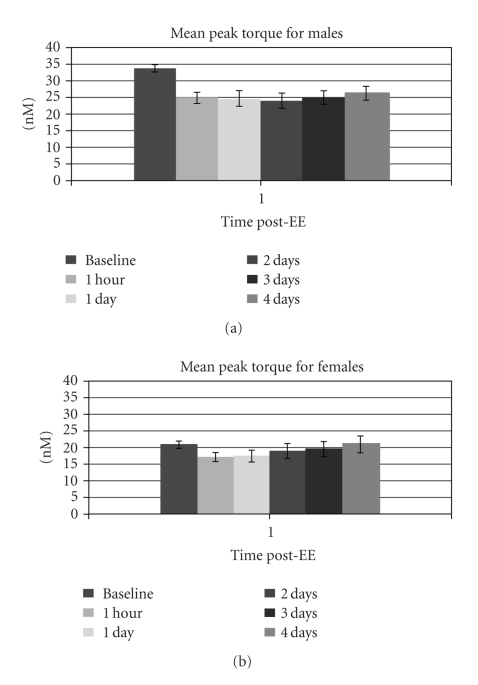
Plot of mean pain intensity rating (scale 0–100) during unloaded movement through full range of motion of the elbow versus serum 25-hydroxyvitmamin D (25OHD) concentrations (nmol/L) in female participants (*N* = 21). Pain rating obtained 2 days after eccentric exercise protocol; 25OHD serum samples obtained within one week of exercise. Linear regression *R*-squared value = 0.07, *P* *value* = .24. Plot, *R*-squared, and *P value* are representative of other pain and muscle strength variables examined in both sexes.

Linear regression of pain measures against serum 25(OH)D showed that vitamin D status did not predict either of the pain outcomes for males or females, at any time after EE. Additionally, vitamin D status did not predict muscle strength expressed as percent of baseline strength. Table 2 lists the results of these regression analyses. To better illustrate this, we plotted (Figure 3) the mean pain intensity rating (scale 0–100) during unloaded movement through full range of motion of the elbow versus serum 25OHD concentrations in female participants (the plot for males was not different).

## 4. Discussion

To further clarify the importance of vitamin D to muscle, this study examined the association of vitamin D status and exercise-induced muscle pain and weakness in healthy people. We tested the hypothesis that lower serum concentrations of 25(OH)D are associated with greater muscle weakness and pain after eccentric exercise in young, healthy volunteers. Our results, however, did not confirm this hypothesis.

The pattern of elbow flexor strength after eccentric exercise was as expected, with a decrease in strength in the 48-hour period after eccentrics, followed by a gradual increase in torque values. Vitamin D status did not predict this outcome at any time point after eccentric exercise, nor did it predict baseline strength. Although the basis for the lack of effects observed in our study is unknown, we offer two possible explanations: age- or genotype-related differences in muscle VDR, or vitamin D threshold effects in muscles may be lower than other tissues.

The number of VDRs present in skeletal muscle declines with age [23]; thus, strength in young adults, such as the participants in this study, may be less sensitive to vitamin D insufficiency because of the abundance of VDR in their myofibers. Moreover, specific VDR polymorphisms are associated with variations in muscle strength [20, 24, 25]. No study has yet investigated the interplay in muscle between vitamin D status and VDR variants. It is possible that individuals with certain VDR genotypes may be more sensitive to vitamin D insufficiency or may respond differently to supplementation as is the case in bone [26] and prostate [27]. 

Heaney has written extensively on the threshold effect of vitamin D in intestinal calcium absorption and calcium homeostasis outcomes; results from these studies put the threshold concentration of serum 25(OH)D at *∼*80 nmol/L [28]. In contrast, Bischoff-Ferrari et al. based a vitamin D threshold estimate on the risk of falls and fractures, which partly reflects muscle function. This inquiry put the threshold concentration of serum 25(OH)D at *∼*40 nmol/L [29]. Two recent studies investigating the association between vitamin D status and muscle strength in young girls also support a lower threshold for muscle function outcomes. Foo et al. [30] found greater handgrip strength in girls with lower serum 25(OH)D concentrations, and Ward et al. [31] reported that vitamin D status was positively associated with lower body muscle power and force in adolescent girls. The mean serum 25(OH)D concentrations were 34 and 21 nmol/L, respectively, for the cohorts in these studies, in contrast to *∼*77 nmol/L for our participants. If the threshold for improvement in muscle function is indeed near 40 nmol/L, the association of improved muscle strength and serum 25(OH)D would only be observed in groups with poorer status. Further investigation of the vitamin D threshold effect as it pertains to mechanisms of hypertrophy following exercise training is required; and indeed, it may point to improved response to therapeutic exercise by patients from populations at greatest risk for vitamin D deficiency, especially those with darker-pigmented skin or those who are not given regular access to sunlight (e.g., hospitalized inpatients or nursing home residents).

Although there is a clear link between low vitamin D status and the pain of osteomalacia, there are no data available for a role of vitamin D in the delayed pain associated with eccentric exercise. In our study, the only known attempt to link the two, participants' ratings of pain intensity and unpleasantness were collected during unloaded movement; our results showed that serum 25(OH)D concentrations did not predict either of the pain measurements. 

Despite our belief that vitamin D status would be negatively associated with muscle pain and weakness, it is possible that a positive relationship exists. At least some of the ensuing pain following eccentric exercise is thought to be caused by an inflammatory response which is essential for muscle tissue remodeling and hypertrophy [32]. This inflammatory response is quite similar to the initial nonspecific immune response to microbial challenge [33, 34]. In its immunomodulatory role, vitamin D enhances this portion of the immune response [35]. It is therefore conceivable that adequate vitamin D status may support a robust inflammatory response to eccentric exercise and thus contribute to a high level of delayed soreness. Furthermore, the active form of vitamin D, 1,25(OH)_2_D, influences the differentiation and proliferation of muscle cells [5]. When those actions are considered along with encouragement of the nonspecific immune response, it appears possible that postexercise hypertrophy could indeed be improved with adequate vitamin D. The logical question, consequently, is what serum 25(OH)D concentration *is* adequate for an optimal postexercise inflammatory response? Just as discussed for the muscle strength outcomes, it may be that the vitamin D threshold for eccentric exercise-associated pain is relatively low, and which might explain why we did not see a relationship between vitamin D status and pain ratings in our participants.

The main limitation of this study is that it involves a secondary data analysis. The exclusion criteria of the primary analysis disallowed anyone with pre-existing pain or muscle disease so it is conceivable that individuals with muscle pain due to hypovitaminosis D would not be included in our sample and may have restricted (especially at the lower end) the range of serum 25(OH)D concentrations observed for participants in our study. We assumed that serum 25(OH)D remained fairly stable throughout the 6-day intervention as it has a serum half-life of ~3 weeks [36]. This assumption may be invalid in the unlikely event that a participant spent a day in the sun after the second day; however, the effect of this event—if it occurred—can be considered rather inconsequential as it takes at least four days to obtain 80% photoconversion of dermal precursors to vitamin D_3_ and even longer to affect 25(OH)D concentrations [37]. 

In conclusion, our data indicates that vitamin D status does not predict outcomes of muscle pain and weakness in young, healthy volunteers after eccentric exercise. Whether the threshold effect of vitamin D in the muscle or some other factor can explain our observed lack of association remains to be elucidated. Regardless, further investigations into exercise training processes are necessary to yield insight into the plausible relationship between vitamin D status and the changes in the benefits and risks of resistance training. There are numerous research groups investigating the phenomenon of exercise-induced muscle damage; serum 25OHD assays could be easily included to yield insight into the effects of vitamin D status within those contexts.

## Figures and Tables

**Table 1:** Participant characteristics (values are means ± SD).

	All	Male	Female
*N*	48	27	21

Age (y)	22.5 ± 3.2	22.5 ± 3.4	22.6 ± 3.2

BMI (kg/m^2^)	25.8 ± 6.8	25.8 ± 4.9	25.9 ± 8.8

Nonhispanic white (%)	83.3%	85.2%	81.0%

Supplement use^a^	2	1	1

Serum 25(OH)D, (nmol/L)^b^	77.4 ± 36.1	78.15 ± 43.4	76.5 ± 24.7

^a^Number of participants using supplements with plausible influence on outcomes, for example, omega-3 fatty acid.

^b^Measures of 25-hydroxyvitamin D were obtained from serum drawn during Session 4, *∼*48-hour posteccentric exercise, representing a single measurement during the study. Serum 25(OH)D concentration ranges for all, males, and females were 19.7–269.3, 32.4–269.5, and 19.7–111.3 nmol/L, respectively.

**Table 2:** Regression analyses of serum 25OHD concentrations versus pain and strength outcomes in participants two days after undergoing the eccentric exercise protocol.

	Males		Females	
Outcome	Range of *R* ^2^ values	Range of *P* values^a^	Range of *R* ^2^ values	Range of *P* values^a^
Isometric peak torque	0–0.02 (25–27)^b^	0.48–0.83	0–0.04 (20-21)	0.41–0.90

Isometric peak torque as % baseline	0–0.03 (25–27)	0.43–0.87	0–0.12 (20-21)	0.12–0.97

Pain intensity during movement	0–0.02 (26-27)	0.42–0.95	0.02–0.05 (20-21)	0.14–0.59

Pain unpleasantness during movement	0–0.01 (26-27)	0.61–0.88	0.02–0.07 (21)	0.23–0.65

^a^No regressions were statistically significant at *P* = .05.

^b^Range of sample size in parentheses.
